# Magnetically Counting Hand Movements: Validation of a Calibration-Free Algorithm and Application to Testing the Threshold Hypothesis of Real-World Hand Use after Stroke

**DOI:** 10.3390/s21041502

**Published:** 2021-02-22

**Authors:** Diogo Schwerz de Lucena, Justin Rowe, Vicky Chan, David J. Reinkensmeyer

**Affiliations:** 1 John A Paulson School of Engineering and Applied Sciences, Harvard University, Cambridge, MA 02138, USA; 2 CAPES Foundation, Ministry of Education of Brazil, Brasilia 70040-020, Brazil; 3 Flint Rehabilitation Devices, Irvine, CA 92614, USA; jrowe@flintrehab.com; 4 Department of Mechanical and Aerospace Engineering, Department of Anatomy and Neurobiology, University of California Irvine, Irvine, CA 92697, USA; vchan2@uci.edu (V.C.); dreinken@uci.edu (D.J.R.)

**Keywords:** wearable sensing, IMU, hand movement, dexterity, rehabilitation, stroke

## Abstract

There are few wearable sensors suitable for daily monitoring of wrist and finger movements for hand-related healthcare applications. Here, we describe the development and validation of a novel algorithm for magnetically counting hand movements. We implemented the algorithm on a wristband that senses magnetic field changes produced by movement of a magnetic ring worn on the finger (the “Manumeter”). The “HAND” (Hand Activity estimated by Nonlinear Detection) algorithm assigns a “HAND count” by thresholding the real-time change in magnetic field created by wrist and/or finger movement. We optimized thresholds to achieve a HAND count accuracy of ~85% without requiring subject-specific calibration. Then, we validated the algorithm in a dexterity-impaired population by showing that HAND counts strongly correlate with clinical assessments of upper extremity (UE) function after stroke. Finally, we used HAND counts to test a recent hypothesis in stroke rehabilitation that real-world UE hand use increases only for stroke survivors who achieve a threshold level of UE functional capability. For 29 stroke survivors, HAND counts measured at home did not increase until the participants’ Box and Blocks Test scores exceeded ~50% normal. These results show that a threshold-based magnetometry approach can non-obtrusively quantify hand movements without calibration and also verify a key concept of real-world hand use after stroke.

## 1. Introduction

Wearable sensing systems are increasingly being used for at-home monitoring of health-related parameters [1]. There are many systems available for providing feedback about the amount of movement of the lower extremities (i.e., step counters and pedometers [2]), but there are relatively few available for providing feedback about the amount of movement of the upper extremities (UE). UE measuring systems would be of use for a wide range of healthcare applications, including orthopedic applications such as monitoring overuse injury or recovery from hand surgery, and neurologic applications such as promoting hand rehabilitation after stroke, cerebral palsy, or spinal cord injury.

Current systems for measuring UE activity typically rely on wrist-mounted accelerometry. The majority of this work has focused on quantifying reduced hand use of adults who have experienced a stroke [3,4,5,6,7,8,9]. Although some commercial wrist accelerometry systems use proprietary algorithms to produce “UE counts”, a common published approach is to count an arm movement every time the wrist acceleration exceeds a threshold, possibly with a weighting factor that scales the count based on the magnitude of the acceleration peak [3,10]. Studies have shown a strong correlation between the ratio of the activity of the more and less affected limb with clinical UE assessment scores [5,9]. Additional information about movement can also be extracted using other kinematic metrics, such as jerk asymmetry, and may relate to the quality of movement [11,12].

Wrist accelerometry has several key limitations, however. First, accelerometers worn on the wrist measure acceleration of the whole body, which is a combination of wrist, arm, trunk, and lower extremity movements [10,13]. This provides a source of noise that is difficult to filter out when attempting to isolate the amount of UE movement. Further, although wrist accelerometry does well at detecting functional activity that involves use of the arm, it is poor at detecting functional activity that mainly depends on hand movement without arm movement [14]. Thus, there is a need for wearable technologies that can isolate and quantify the most distal components of UE movement: finger and wrist joint movement.

**Figure 1:**
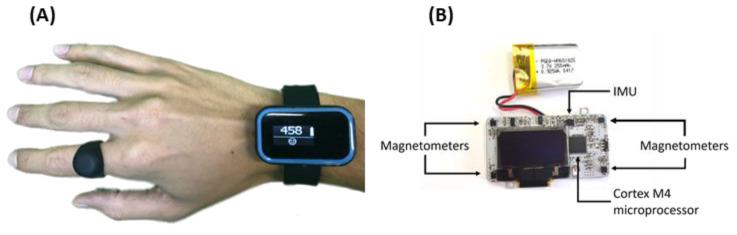
The Manumeter. (**A**) The Manumeter is comprised of a sensing wrist band a ring containing a permanent magnet. (**B**) The Manumeter sensing board, with four magnetometers positioned at the corners of the board, an IMU with six degrees of freedom, an OLED display, and a non-ferromagnetic battery. A Cortex M4 microcontroller controls the components.

Hand activity can be quantified using instrumented gloves, goniometers, motion capture systems, and computer vision [15,16,17], but such devices are cumbersome to wear and may be socially unacceptable, restricting their use mainly to the laboratory or to home studies performed by intrepid participants. Liu and colleagues recently proposed the use of a finger accelerometer as a companion to wrist accelerometry and showed correlation to a gold-standard metric of hand use [18]. However, to house and power the electronics, the ring is bulky and further studies are needed to prove user acceptability. In our laboratory, we demonstrated the viability of a device—the Manumeter—to detect finger and wrist movement while looking like jewelry [6,19] (Figure 1). The user wears a small permanent magnet as a ring, and a sensing/logging wristband uses an array of magnetometers to detect changes in the magnetic field as the ring moves. We developed an algorithm that uses these changes with a neural network to estimate the wrist and finger joint angles. We characterized the accuracy of this algorithm and showed suitability of the Manumeter for at-home use [6,14,19]. However, estimating joint angles in this way requires subject-specific calibration for different hand sizes and computationally demanding algorithms. In addition, pilot users commented that the total angular distance traveled by the joints was not an intuitive measure of their amount of hand use.

To overcome these issues, we developed a novel hand-movement counting algorithm for the Manumeter called HAND (Hand Activity estimated by Nonlinear Detection). We had two main goals in developing the HAND algorithm. First, we desired that the counts of movement be relatable, similar to steps with a pedometer, where one step is a well-defined, easy-to-understand event. Second, we desired that the algorithm be free of user-specific calibration, and, in particular, insensitive to hand size. These two characteristics were desirable for creating a practical device not only capable of quantifying hand use, but also capable of providing real-time, intuitive, quantitative feedback.

The HAND algorithm achieves these goals by thresholding the real-time change in a magnetic field—a computationally simple approach. As we show below, by choosing a threshold close to the noise level but requiring multiple samples to be above that threshold, the algorithm counts movements that are both slow and fast, and small and large (similar to how a pedometer counts different types of steps). We also show that this approach assigns counts in a way that is fairly insensitive to hand size. Thus, it does not require user-specific calibration.

In this paper, we first describe the design of the HAND algorithm, and then experiments we ran to optimize its parameters. We then describe a series of experiments with unimpaired adults and persons with hand impairment after a stroke, both in the laboratory setting and at home, to characterize and validate HAND counts. The home experiments focused on testing the so-called “Threshold Hypothesis” for hand use after stroke, which posits that persons with hemiparesis after stroke rarely use their impaired hand unless the functional capability of their impaired hand (as measured in the clinic) exceeds a threshold [20]. Schweighofer et al. generated this hypothesis based on analysis of subjective, self-estimation of daily hand use; here we test it with HAND counts measured objectively during daily life.

## 2. Materials and Methods

### 2.1. The Manumeter

The Manumeter (Figure 1) consists of four magnetometers (LIS3MDL) located 25 and 42 mm from each other on the corners of a board enclosed in a rectangular watch-like device (sensing range of ±4 gauss with a 16-bit resolution), and a six-degrees-of-freedom inertial measurement unit (IMU, LSM6DSL, accelerometer range set to ±4 G and gyroscope range set to ±500 degrees per second, both with a 16-bit resolution). Time and date are managed by a real-time clock (PFC2123).

A system-on-a-chip (NRF52, Nordic Semiconductor) with an ARM Cortex M4 CPU and wireless capabilities is used to collect and store the data from the IMU and magnetometers into an on-board 4 GB flash memory (MT29F4G01ADAGDWB-IT:G TR) at 52.6 Hz. The data can later be transferred using the Enhanced Shockburst wireless protocol to a secondary board carrying an SD card. The Manumeter also has an OLED display, status LED, and a push-button available, but they were not used for the experiments presented in this paper. The Manumeter is powered with a non-ferromagnetic battery (PGEB-NM651825-PCB) with 250 mAH rated capacity that lasts for about 24 h in continuous use or two weeks in standby mode. The companion ring (i.e., the magnetic ring), worn on the index finger, is made of silicone and holds an N52 grade neodymium magnet disk of 12.7 mm diameter and 3.18 mm thickness.

### 2.2. Removing the Effects of Earth’s Magnetic Field

Because the magnetic field of the Earth is approximately constant over small distances and the strength of the magnetic field of the magnet in the ring drops inversely to the square of the distance, we can remove the effects of the magnetic field of the Earth by taking the differential signal between the magnetometers located closer to the magnetic ring and the ones further away. We used the average of the differential of the two magnetometers on the left side of the housing and the differential of the two magnetometers on the right side of the housing. Because we took an average of the two sides, the below algorithm works the same way when the device is placed on either hand. For the remainder of this paper, we refer to this average as the “differential reading”. The differential reading ranges in magnitude up to about 0.12 milligauss, which can be compared to Earth’s magnetic field, which is about 0.5 gauss in California (and ranges from 0.25 to 0.65 gauss across the globe).

### 2.3. The HAND Count Algorithm

To develop a threshold-based algorithm, we first analyzed the noise in the magnetic field sensing. We quantified the number of sequential changes of the differential reading in the same direction in each axis when the device was at rest; i.e., looking at one axis, how many times in a row did the differential reading increase or how many times in a row did it decrease when there was no movement of the finger-worn magnet? Our goal was to characterize this noise so we could assign a hand movement count when the measured differential reading deviated from this background noise.

**Figure 2:**
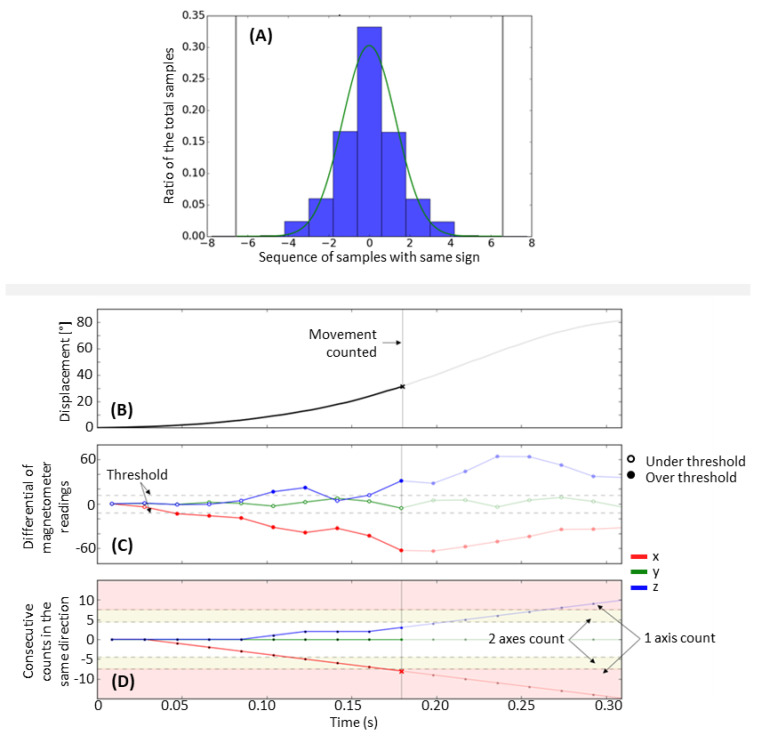
Overview of the Hand Activity estimated by Nonlinear Detection (HAND) count algorithm (**A**): Distribution of consecutive changes in the differential reading for all axes combined when the magnetic-sensing wrist-band rested on a table away from the ring magnet. 0 means that two consecutive differential readings had different signals (e.g., one positive, one negative), 1 means that there was 1 consecutive differential reading with the same signal, 2 means that there were two consecutive differentials with the same signal, etc. The grey vertical line shows three standard deviations from the mean. (**B**–**D**) Show an example of the HAND algorithm applied to data acquired during a wrist extension with the hand open (**B**), displacement in degrees of the wrist during an extension movement; (**C**) differential reading for each of the axes (x, y, z); each circle is one data point; dashed lines are the threshold of minimum (positive or negative) change to be counted; empty circles are not counted (within thresholds); filled circles are counted; (**D**) current count for each of the axes; yellow shaded area is the threshold for two axes together—when two axes are in the yellow area, it is counted; red shaded area is the threshold for one axis to be counted (in this example, the *x*-axis reached the threshold and the movement was detected). For this figure, LPcutoff = 16 Hz and threshold = 16 differential readings, which results in the standard deviation of 1.2 for the distribution of sample direction count. SDs (2 axes) and + SD (1 axis) were set to 4 and 3, respectively.

The number of same-direction changes followed a normal distribution with an average near zero (Figure 2A). The HAND algorithm takes advantage of this characteristic by counting the number of times the differential reading changes in the same direction for two axes with at least a small minimum strength, and then considering a hand movement to have occurred when this count exceeds a threshold. The algorithm also monitors when only one axis changes in the same direction for a (different and larger) minimum number of samples. This method can be interpreted as a peak detection algorithm using a small threshold; a method that has been successful in wrist accelerometry. However, this method also takes advantage of the differential reading’s short sampling period, which is approximately an order of magnitude shorter than a typical hand movement (which is 200–700 ms). This allows us to require multiple samples above the threshold before signaling that a movement has occurred.

Given this algorithm structure, there were four parameters to be selected: (1) LPcutoff, which is the low-pass filter cutoff frequency for a second-order Butterworth filter that is applied to the differential readings; (2) the threshold, which is the minimum absolute value needed for a differential change in the differential reading (i.e., a “sample”) to be counted as positive or negative for that specific axis (if smaller than the threshold, the sample is ignored); (3) SDs (2 axes), which specifies the minimum sequence length required for samples in the same direction on two axes. This parameter is a scale factor (constrained to be an integer and greater than zero) applied to the standard deviation of the distribution of the same-direction sample sequence length (Figure 2A). Note, this distribution (and thus the standard deviation of the distribution) depends on the LPcutoff and threshold parameters, so we calculate the standard deviation after first applying the filter and the threshold to a data set with no hand movement (see “arm-only exercise” below). The scaled standard deviation is rounded to the closest integer and used as the minimum number of same-direction samples required on two axes at the same time to signal that a hand movement has occurred. For example, if the standard deviation of the same-direction sequence length is measured to be 1.5 and we set SDs (2 axes) to 4, at least two axes needed to have 6 or more samples (all positive or all negative) with an absolute value greater than the threshold parameter to be counted as a hand movement; (4) + SDs (1 axis) is the number of additional same-direction samples (i.e., over the number indicated by SDs (2 axes)) that we require to occur on a single axis (instead of two axes) to signal that a hand movement has occurred. Expressing it as the number of additional samples makes it explicit that the algorithm applies a more stringent criterion when looking at only a single axis. After counting a hand movement, the algorithm is reset when there is a change in signal direction of the axis that triggered the count or after two seconds have elapsed since the last count. Figure 2B–D shows an example of the algorithm applied to wrist extension data. Upper extremity activity counting using accelerometry

To compare the HAND algorithm to a widely used measure of UE activity, we used a variation of the wrist accelerometry algorithm proposed in [3,10]. Using data acquired from the accelerometers in the Manumeter, we calculated the sum of the absolute change from sample to sample over a running window of 0.25 s (about 13 samples). We applied a peak detection algorithm to the running sum with a threshold of 0.33 g and limited possible detection to a maximum of 4 peaks per second, where each peak was defined as an “activity count”.

### 2.4. Experimental Protocol

To evaluate the performance of the HAND algorithm, eight unimpaired volunteers (six males and two females, mean age 26.1 ± 3.0 SD years) performed hand and arm exercises in the laboratory. In addition, we recruited two groups of participants with hemiparesis in the chronic phase after stroke (see Table 1). All participants provided written consent and the UC Irvine Institutional Review Board approved all experiments.

The first group of participants with a stroke first completed a visit to the laboratory where demographic information was collected. A physical therapist fitted and donned the Manumeter and the magnetic ring on the affected wrist and index finger of the impaired hand, then performed standard clinical assessments (see below). On leaving the laboratory at the completion of the assessments, participants were instructed to wear the device throughout the rest of their day, continuing with their normal daily routine until going to take a shower or turning in to sleep, when they were instructed to remove the device. They brought the device back or mailed it back the next day. We chose to avoid asking the participants to wear and remove the device to ensure that they did not swap hands or misplace the device during the data collection. The participants were also asked to log their activities during the day and the time they removed the device, which we used to validate the time stamping of the data.

The second group of participants who had experienced a stroke visited the lab two more times (these visits were part of a separate study looking at the effect of real-time hand feedback on motor recovery, the results of which will be presented in another paper). The second visit was 4 weeks and the third visit 4 months after the first visit. The clinical assessments were again performed during all visits while wearing the Manumeter. In the last visit, participants performed a 1-minute walk test. Once again, participants were asked to wear the Manumeter at home during their normal daily activities.

For both groups, participants met the following criteria: (1) 18 to 80 years of age; (2) experienced one or multiple strokes at least six months previously; (3) Fugl-Meyer Upper Extremity Score < 60 (out of the maximum of 66); and (4) absence of moderate to severe upper limb pain (<3 on the 10-point visual-analog pain scale). Participants with implanted pacemakers were not allowed in the study for safety reasons concerning the magnetic ring.

### 2.5. Upper Extremity Exercises

**Figure 3:**
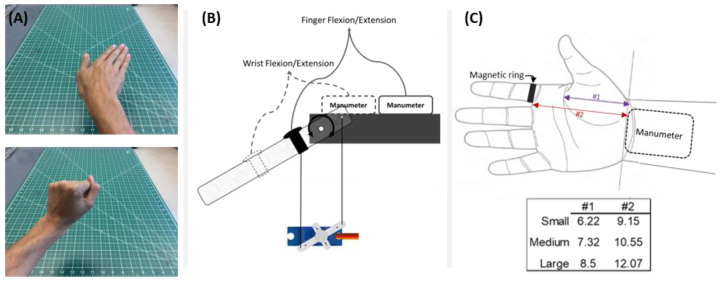
Overview of experimental methods used to produce known hand counts. (**A**): Screenshots of movie showing two of the desired hand postures that participants had to mimic during the hand-only exercises. (**B**) Apparatus used for movement emulation. For wrist flexion and extension, the wrist joint was emulated, and the acrylic piece represented the hand and index finger together. For finger flexion and extension, the metacarpo-phalangeal (MCP) joint of the index finger was emulated and the acrylic piece represented the index finger only. (**C**) Measurements used for defining the emulated hand sizes in centimeters. The Manumeter was aligned with the wrist crease baseline and the magnetic ring was placed touching the interdigital folds of the index and middle finger. Dimension #1 is from the wrist crease baseline to the intersection of the distal transverse palm crease with the ulnar edge of the palm. This is the best approximation to the center of rotation for the MCP joint of the index finger. Dimension #2 goes from wrist crease baseline (which closely aligns with the center of rotation of the wrist) and the interdigital folds of the index and middle finger.

Unimpaired participants and both groups of individuals with a stroke performed a set of UE exercises, which we call the “hand-only” and “arm-only” exercises. For the hand-only exercises, subjects sat on a wooden chair with their arms on the chair’s armrest. A tablet was placed at a comfortable distance in front of the subject. The subjects were instructed to mimic the hand postures prompted on the tablet. To perform the change in hand posture, participants were asked to perform one single, smooth movement of the hand. The postures were composed of 5 wrist positions: neutral, flexed, extended, ulnar deviated, and radially deviated, each of them with fingers flexed or extended, for a total of 10 hand postures. Each posture was prompted 5 times for 2 s for a total of 50 prompts. Figure 3A shows two sample hand postures presented to the participants. The accuracy of the Manumeter was calculated as the percentage difference with respect to the total number of executed hand postures (i.e., 50).

For the arm-only exercises, subjects donned a splint holding the wrist and the finger in a neutral position and stood. A tablet was again placed at a comfortable distance in front of the subject. Starting with their upper arm next to the body, the elbow flexed at 90 degrees, and the hand in front of the body, participants were asked to perform an arm movement in the direction presented on the screen and come back to the original arm position (two movements per direction). Five directions were used: front, up, right, left, and rotation of the wrist (supination). The directions were presented as text and graphically using arrows. A new direction was prompted every 2 s. Each direction was presented 20 times for a total of 200 arm movements (20 × 5 directions × 2 movements/direction). Any hand counts during this exercise were considered false positives (because the wrist and fingers were held in a splint that prevented their movement), and the “cross-talk” error was calculated as the percentage of the arm movements there were counted as hand movements.

### 2.6. Clinical Evaluations

Participants with a stroke were evaluated by an experienced physical therapist on a well-established clinical measure of UE function, the Box and Blocks Test (BBT) [21], and a well-established measure of UE impairment, the Fugl-Meyer Upper-Extremity Subscale (FMUE) [22]. They also walked for one minute while wearing the Manumeter. Participants were instructed to walk at their normal speed in a long hallway while two people counted the number of steps they took. No instructions regarding the Manumeter, hand movements, or finger movements were given during these tests.

### 2.7. Algorithm Characterization with a Robotic Simulator

To test the HAND algorithm’s ability to count movements for different hand sizes, movement speeds, and movement amplitudes, we set up a robotic testbed to emulate wrist and finger movements. The testbed had a servo motor connected to an acrylic piece using two strings (Figure 3B). A magnetic ring was placed on the acrylic piece at different distances depending on the hand size and movement to be emulated. The Manumeter was aligned with the emulated wrist (Figure 3C). 

This setup allowed us to simulate two types of movement: (1) wrist flexion/extension with the metacarpo-phalangeal (MCP) joint extended, and (2) finger flexion/extension with the wrist in a neutral position (palm of the hand parallel with the forearm). For wrist flexion/extension, the servo motor emulated the wrist joint and the acrylic piece represented the extended hand and index finger. The distance from the Manumeter to the magnetic ring followed dimension #2 in Figure 3C. For the finger flexion/extension, the servo motor emulated the MCP joint of the index finger and the acrylic piece represented the index finger. The distance from the Manumeter to the emulated joint followed dimension #1 in Figure 3C and the distance from the Manumeter to the ring was kept as dimension #2.

We compared the performance of the algorithm for three simulated hand sizes (small, medium, and large) based on [23]. The small hand used the measurements of the 5th percentile of women’s hand, the medium used the average between the median hand sizes of men and women, and the large hand used the 95th percentile of the men’s measurements as shown in Figure 3C. The movements ranged from 5 to 90 degrees in size with 5-degree increments. The movement velocity followed a bell-shaped profile with absolute peak angular velocities ranging from 20 to 600 degrees/second with increments of approximately 10 degrees/second. In total, 986 movements were performed for each hand size and each movement type. These movements were then categorized as slow (<200 degrees/second), medium (200 to 400 degrees/second), and fast (>400 degrees/second) based on average absolute wrist movement speed presented in [24].

## 3. Results

### 3.1. Selecting the HAND Algorithm Parameters

**Figure 4:**
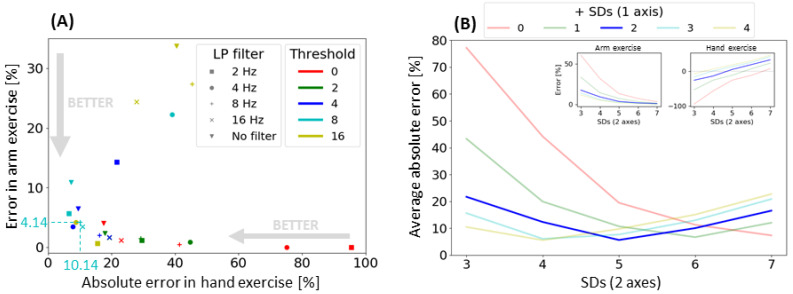
HAND algorithm parameter exploration. (**A**) Counting error for the arm-only exercise and the hand-only exercise for different thresholds (represented by the colors) and cutoffs (represented by the markers) for the low-pass filter. For the arm-only exercise, the error is calculated as the percentage of hand counts to the number of arm-only movements averaged across subjects. For the hand-only exercise, the error is calculated as the absolute difference between the number of hand counts as a percentage to the number of actual hand movements performed averaged across subjects. For both exercises, smaller errors are preferred. The errors for the selected parameters (LP filter cut-off = 8 Hz, threshold = 8) are highlighted. (**B**) Average error for arm-only and hand-only exercise of the hand count algorithm for different SD parameter 2 axes (*x*-axis) and number of extra SD for 1 axis (represented by the color) using LP filter cut-off = 8 Hz, threshold = 8. The two inner figures show the percentage error for arm (left) and hand (right) exercises. The selected SDs are 5 for two axes plus 2 SDs for one axis.

A total of eight unimpaired participants completed the arm-only exercises and hand-only exercises while wearing the Manumeter on the left and right arm. To explore the effects of the LPcutoff and threshold parameters, the SDs (2 axes) and + SDs (1 axis) were defined as 5 and 2, respectively, based on experience with initial hand-tuning. We applied the hand count algorithm to the arm exercise and hand exercise data using a combination of no filtering; 2, 4, 8, and 16 Hz for the LP Filter; and 0, 2, 4, 8, and 16 to the threshold. The percentage errors for arm and hand exercises are presented for each pair of LP cutoff and threshold (Figure 4A). As can be seen, we observed a tradeoff between counting false positives during arm exercise and miscounting during hand exercise. In a range of LPcutoff (4 to 16 Hz) and thresholds (4 to 8 differential readings), both errors are minimized. Based on these results, we selected 8 Hz for the LPcutoff and 8 for the threshold parameter, which corresponded to an average error of 4.14% for arm exercise and 10.14% for hand exercise.

Using these selected parameters, we explored the effects of SDs (2 axes) and + SDs (1 axis) on the algorithm performance. We calculated the average absolute errors for the arm and hand exercise for each combination of parameters (Figure 4B). We obtained the overall best performance with 5 and 2 for SDs (2 axes) and + SDs (1 axis), respectively. For the remainder of the results presented here, we set the parameters of the algorithm to LP Filter = 8 Hz, threshold = 8 differential readings, SDs (2 axes) = 5, + SDs (1 axis) = 2.

### 3.2. Algorithm Characterization: Effect of Amplitude and Speed

**Figure 5:**
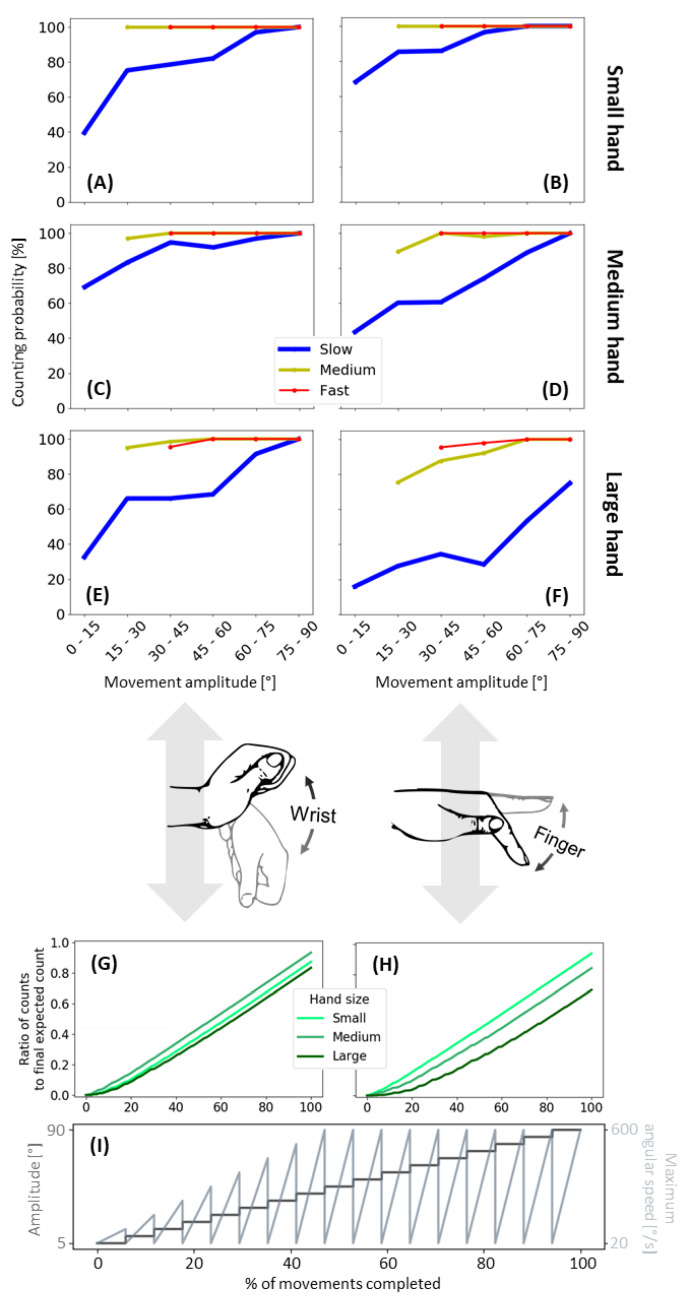
Results from the robotic simulation of wrist and finger movements. (**A**–**F**) Probability of counting emulated movements. (**top to bottom**) Shows probabilities for the three hand sizes: small, medium, and large; and (**right to left**) Shows probabilities for the emulated exercise types: wrist flexion and extension, and finger flexion and extension. For each hand size and exercise type, absolute maximum angular speed of the movement was used to classify it as slow, medium, and fast movement. The probabilities were calculated as the number of hand counts over the number of movements. (**G**,**H**) Show the number of counts over the total expected counts using emulated data for (**G**) wrist flexion and extension, and (**H**) finger flexion and extension. Bottom plot (**I**) shows the progression of the emulated movements that increased in amplitude over time sweeping through a range of speeds each amplitude. The range of speeds changes for the different amplitudes due to limitations on the maximum angular acceleration of the motor.

To understand the effects of movement amplitude and speed on the accuracy of the HAND algorithm, we used a robotic device to emulate two types of hand movements: wrist flexion/extension and finger flexion/extension. Both types of movements were executed ranging from 5 to 90 degrees in amplitude and 20 to 600 degrees/second for absolute peak angular speed. The emulation started with small and slow movements, going through the range of angular velocities for each amplitude (Figure 5I). Due to limits on angular acceleration, the maximum peak angular speed was reduced for smaller movement amplitudes—similar to what is seen with human wrist movement [24]. The ratio of counted wrist and finger movements to the number of known movements was calculated for three different hand sizes (Figure 5G,H). A line of unity slope was expected if all movements were counted. Instead, the slope was reduced for movements smaller than 20 degrees of amplitude. Out of a total of 986 movements for each emulated movement and hand size, the final accuracy for wrist flexion and extension was 87%, 93%, and 83% for the small, medium, and large hand, and for finger flexion and extension was 94%, 84%, and 70%, respectively, for the different simulated hand sizes. Thus, the average accuracy across speeds, movement amplitudes, and simulated hand sizes was 85%.

To understand the effect of movement speed, we analyzed the counting probability for slow, medium, and fast movements separately (Figure 5A–F). For slow movements, and in particular, for finger flexion and extension, increases in hand size decreased the algorithm’s performance. This was likely due to the smaller total distance traveled by the magnetic ring for the finger movement compared to the wrist movement. For all cases, lower probabilities were found during slow movements with a steady decrease for slower movements (below 20% in the worst-case scenario). Due to physical constraints of the emulation system, movement amplitudes were limited to 90 degrees; however, for all movement types, movement speed, and hand sizes (except for large hand, slow movements for finger flexion and extension) the algorithm accurately counted all of the movements for the largest amplitudes (75 to 90 degrees) indicating that the same would be true for larger movements. For large hand and slow movements of the finger, the algorithm had a counting probability of 78% for the largest amplitudes.

### 3.3. Accuracy in Counting a Known Amount of Hand Movements

A total of eight unimpaired participants and nine stroke survivors performed the hand-only exercises with a total of 50 hand movements. Stroke survivors performed the exercise only with their impaired hand. Two of the stroke survivors could not perform the exercise without assistance and were allowed to use their unimpaired hand to help complete the movements. Unimpaired participants performed the exercise twice, one with each hand. Unimpaired participants also performed the arm-only exercises with both arms using a splint which ensured that hand movement was not possible.

**Figure 6:**
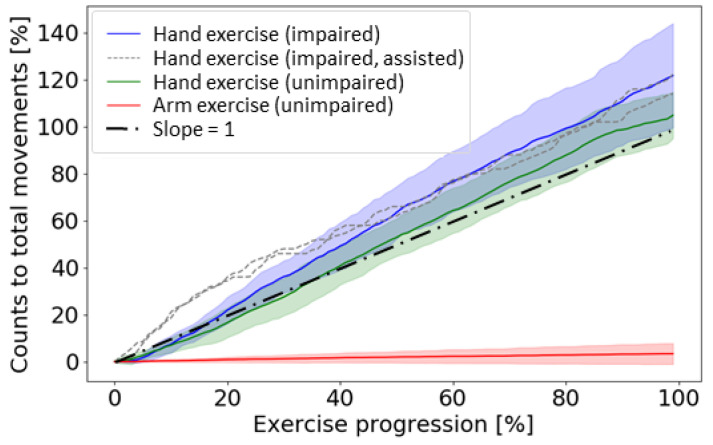
Hand counts for hand-only and arm-only exercises performed in the laboratory plotted as a function of exercise progression. Subjects were either impaired (due to hemiparesis after a stroke) or unimpaired. Two subjects in the impaired group could not perform the hand-only exercise without assistance of their unimpaired hand and are presented with the dashed lines. The impaired, assisted subjects had an average count accuracy of 82% for hand-only exercise. The remainder of the impaired subjects (*n* = 7) performed the exercise without assistance and had an accuracy of 78% for the hand-only exercise. Unimpaired subjects (*n* = 8) had a count accuracy of 95% for the hand-only exercise. Perfect counting for the hand-only exercise would be a line with a slope of 1. Only unimpaired subjects performed the arm-only exercise with 6% of crosstalk. Shaded areas show 1 SD.

For the hand exercises, in which 50 hand movements were expected, the unimpaired participants had on average 52.4 ± 4.9 hand counts, an overcount error of 4%. The stroke survivors had an average hand count of 60.9 ± 11.1 for the seven participants who could perform the movements with no assistance, and 61 and 57 for the two subjects that self-assisted their hand movement. The overcounting error was thus about 20% considering all impaired participants. For the arm exercises, in which no hand counts were expected, participants had an average of 6.8 ± 8.8 hand counts after 200 arm-only movements (Figure 6), and an error rate of 3.4%.

### 3.4. Correlation with Clinical Scales of Hand Movement Ability

**Figure 7:**
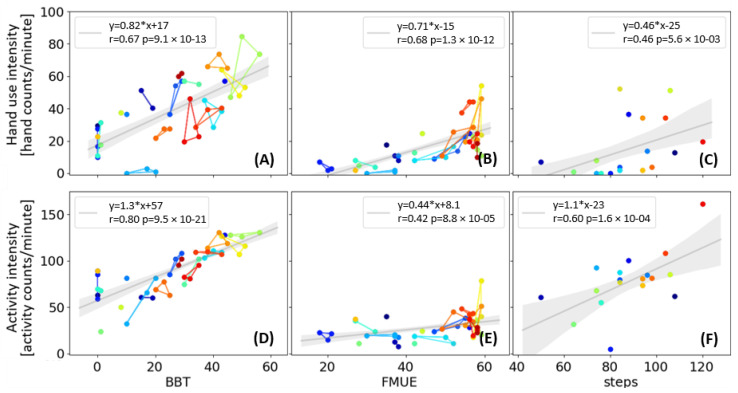
Hand use intensity and activity intensity (**from top to bottom**) during clinical tests (the Box and Blocks Test—BBT, the Fugl-Meyer Upper Extremity Assessment—FMUE, and the 1-minute walking test), (**from right to left**) performed in the lab. Linear fit is presented with the shaded area showing the 95% confidence interval. Each color (connected by lines) represents one subject, and each subject can have one to three samples for each test.

Another way to validate a wearable sensing algorithm is to determine how well HAND counts correlate with clinical measures of upper extremity movement ability. Twenty stroke survivors wore the Manumeter on their impaired arm while performing the Box and Blocks Test (BBT) and the Fugl-Meyer Upper Extremity Assessment (FMUE) up to three times (separate visits), and during a one-minute walk test (Figure 7). The BBT requires participants to pick up, transport over a divider, and drop as many small blocks as they can in 60 s, and thus the BBT score (number of blocks moved) should directly reflect HAND counts. The FMUE progresses from arm-only to wrist and hand test movements; higher scores reflect more ability to move the arm. We analyzed how the participants’ clinical scores on these tests related to the hand counts obtained during the test, and compared it to the “UE activity counts” obtained from the accelerometers in the Manumeter, using a conventional arm accelerometry algorithm. For this analysis, we computed “hand use intensity” and “UE activity intensity” as counts per minute achieved by the participant while actively performing the test.

For the BBT, there was a strong correlation between BBT score and both the hand use intensity (r = 0.67, *p* < 0.01) and UE activity intensity (r = 0.64, *p <* 0.01) (Figure 7A). If the participants with BBT = 0 were ignored, the slope for hand counts with respect to BBT score was 0.9; a value of 1 would be expected if the algorithm assigned one HAND count to each block moved (releasing the block can usually be done with a small movement that the Manumeter did not count). The slope of UE activity counts with respect to BBT score was 1.8 (Figure 7D); a value of 2 would be expected, because for each block transferred, participants needed two movements of the arm.

For the FMUE tests, there were strong correlations between hand use intensity and FMUE score (r = 0.68, *p <* 0.01) (Figure 7B, E). UE activity intensity had a weaker correlation with the FMUE score (r = 0.42, *p <* 0.01). There was a sharp increase in hand counts after FMUE score of 40, which is approximately the score at which individuals experience recovery of hand movement [25].

We also measured hand counts as people walked for one minute to test if walking produced spurious hand counts. Hand intensity was moderately correlated with step counts with a slope of 0.46 (Figure 7C). Wrist-accelerometry-derived UE activity intensity was more strongly correlated with step counts with a higher slope of 1.1 (Figure 7F). Thus, the magnetic sensing approach was less affected by walking than wrist accelerometry, although it was still affected.

### 3.5. Testing the Threshold Hypothesis for Hand Use after Stroke

A recent hypothesis in stroke rehabilitation is that real-world UE use lags clinically demonstrated UE functional capability until UE capability reaches a threshold [20]. That is, participants with a stroke who can achieve some level of hand function in a clinical setting still do not use their hand at home if that level of hand function is too low. Schweighofer et al. originally generated this hypothesis using data of self-reported hand use at home [20]. Here, as a further test of validity of the HAND algorithm, we sought to determine if sensor-measured HAND counts are consistent with the Threshold Hypothesis.

A total of 29 stroke survivors wore the Manumeter at home during their daily activities. The first nine participants wore a previous version of the Manumeter in which data was sampled at 30.3 Hz; however, the same algorithm was used, and selection of parameters was performed in a similar fashion as presented above. These participants only wore the Manumeter for one day, for 9.21 ± 1.75 h. The remainder 20 stroke survivors wore the Manumeter twice, first for 5.88 ± 2.41 h and then for 6.85 ± 1.98 h. Data were lost for three participants in their first visit due to technical problems with the device.

**Figure 8:**
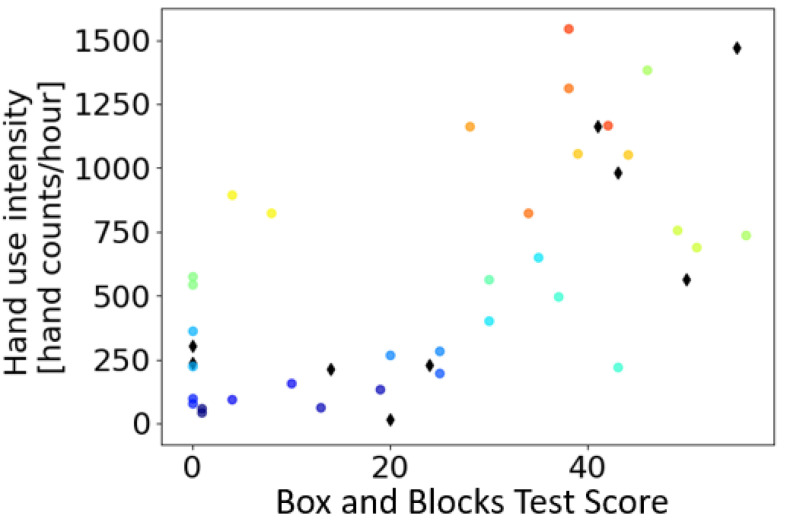
Testing the use capacity Threshold Hypothesis for the stroke-affected hand. Shown is hand use intensity measured at home for 29 individuals with a stroke with different levels of hand functional capacity, as quantified by the Box and Blocks Test (BBT) score. For the circles, each color represents one subject, and each subject can have one to three samples for up to three different days. For the diamonds, each data point is a different subject, and data was acquired with a previous version of the system sampled at 30.3 Hz instead of 52.6 Hz.

Intensity of hand use, calculated as hand counts/hour, was significantly correlated with BBT score (r = 0.64) (Figure 8). Most participants with BBT < 30 were approximately constant in hand use intensity at around 200 hand counts/hour (similar to the intensity measured when one subject did not wear the magnetic ring for several days; see Section 3.6). Then, there was an increase in hand use intensity as participants’ BBT score increased beyond 30 (approximately 50% of a normal BBT score), consistent with the Threshold Hypothesis. However, two of the most-impaired stroke survivors (yellow and green on the left of Figure 8) had relatively high hand use. We note that these two participants were much younger than the remaining participants (18 and 24 years old compared to an average of 63 years old) and attending college.

### 3.6. Quantifying the Effect of Environmental Magnetic Noise on HAND Counts

**Figure 9:**
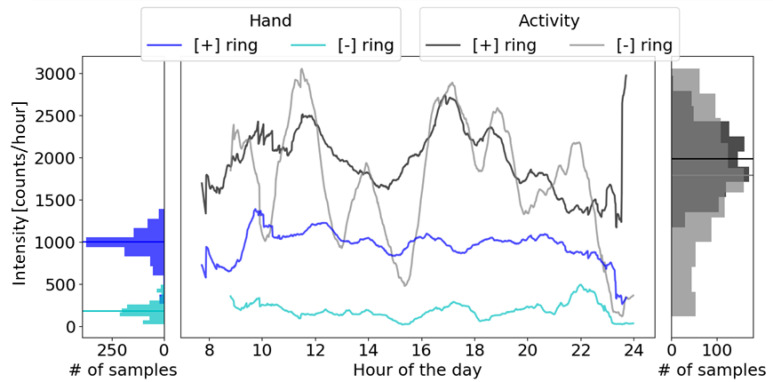
Effect of environmental magnetic noise on HAND counts. Data is from one subject with a stroke (BBT score = 40) who used the Manumeter for 21 days. The subject wore the Manumeter without the magnetic ring ([−] ring) for 6 of the 21 days and with the ring ([+] ring) for the remainder of the days. The HAND counts and UE activity counts (derived from the wrist accelerometer in the Manumeter wristband) were smoothed using a 1-h running window average and are presented in the middle plot as the average across the days with and without the ring. The left and right plots show the distribution of hand and activity counts per hour with a line representing the average intensity. Note that HAND counts dropped to ~200 counts/hour when the ring was not worn, compared to ~1000 counts/hour when the ring was worn (left distributions, mean bars). Activity counts measured with wrist acceleration stayed about the same on average (right distributions, mean bars), whether the ring was worn or not, as expected. The ~200 HAND counts/hour measured without the ring are due to environmental magnetic noise.

One stroke participant (BBT = 40) lost the magnetic ring. We instructed him to keep wearing the wristband until we could send him a replacement in order to understand how environmental magnetic noise, such as that arising from moving past ferrous metal objects and manipulating cell phones, affected HAND counts. He wore the Manumeter at home for 15 days with the magnetic ring and 6 days without the magnetic ring (Figure 9). The number of hand counts without the magnetic ring was on average 18% of the hand counts during the days with the ring, and was about 200 counts/hour. Note that we measured a similar level of UE activity with wrist accelerometry for this participant when he did or did not wear the magnetic ring (Figure 9).

## 4. Discussion

We described the development and validation of a calibration-free, computationally simple algorithm for measuring hand activity in real-time with the Manumeter, a wearable sensor that looks like jewelry (i.e., a watch and a ring). We optimized the parameters for the algorithm to simultaneously reduce errors in counting hand movement in a laboratory-based set of hand exercises, and spurious hand counts from arm-only exercises. We characterized the algorithm performance using a robotic device that emulated wrist and finger movement, finding an overall accuracy of 85%, but reduced accuracy for small, slow movements, and larger simulated hands. Counts of hand movements by unimpaired and stroke participants in the laboratory overestimated the actual count by about 4% and 20%, respectively. Hand counts were still generated erroneously when participants walked, but at less than half the rate as arm activity counts derived from accelerometry. Hand counts measured from the stroke participants were strongly correlated with the scores in the BBT and FMUE clinical assessments. Finally, we calculated HAND counts for stroke survivors at home. We showed that for a range of hand impairment level (BBT score < 30), people with a stroke exhibited a low level of hand use intensity. Hand use rose quickly for participants with BBT score > 30, consistent with the Threshold Hypothesis of hand use after stroke.

### 4.1. Rationale for the HAND Algorithm

A previous algorithm for hand movement counting developed for the Manumeter successfully measured total distance traveled by the finger and was validated for multiple days with no recalibration [19]. However, it required user-specific calibration, which was sensitive to hand size and the positioning of the device on the wrist. The algorithm was computationally complex, and although it had the potential to be embedded in a wrist-worn device, the power requirements were elevated.

A computationally simple thresholding approach has been used for wrist accelerometry to measure upper extremity activity out of the clinic [5,10,11] and we considered applying the same technique to the Manumeter data. However, we observed a lower signal-to-noise ratio with the Manumeter compared to wrist accelerometry and setting the appropriate threshold for different movements speeds and hand sizes would be challenging. Moreover, although it has been successful in estimating upper-extremity activity, the counts obtained with the standard thresholding approach for wrist accelerometry are somewhat difficult for users to relate to, because they represent counts over bins of time rather than specific movements and, in many cases, they are scaled by peak acceleration. Here, we provide a solution to these issues by counting hand movements in a manner analogous to the way a pedometer counts steps.

### 4.2. Factors Influencing HAND Count Accuracy

HAND count accuracy decreased for slow, small movements generated by a custom-built, robotic simulator. However, some of the emulated movements were well below human average speeds. In another study, when unimpaired subjects were asked to make 20-degree wrist flexion/extension movements to targets at “half natural” speed, they made movements with a peak velocity of 200 degrees/second [24]. In our analysis, movements with peak velocity below 200 degrees/second (down to 20 degrees/second) were classified as slow movements and were the main source of undercounting. Note that, even though there is a decline in accuracy with an increase in hand size, the hand sizes emulated here are on the extremes expected for humans (large hand in the 95th percentile for men), so this error can be seen as a worst-case estimate for the algorithm performance.

We further investigated the algorithm accuracy through in-lab experiments in which participants made a known number of hand movements by following a visual prompt. The HAND algorithm overcounted by 4% for unimpaired participants and 20% for stroke survivors. The overcounting may have been due to participants sometimes making multiple movements when changing from one instructed posture to the next. This was particularly prominent for stroke survivors who sometimes appeared to decompose movements into segments, a well-documented phenomenon following stroke [26]. An interesting direction for future research is to determine how measures of smoothness, in addition to clinical measures of spasticity and abnormal synergies, relate to count accuracy.

Pedometers have been reported to have comparable accuracy (70–90%) when measuring distance traveled outside of the clinic [27]; therefore, we expect utility at this level of accuracy. For applications of wrist accelerometry for measuring upper extremity activity of stroke survivors, the variability in the test-retest has been shown to be 39% for raw counts and 3% for threshold-filtered counting [3]. Comparison with wrist accelerometry is important because it is the current standard for upper extremity activity measurement, although we also note these methods focus on arm movement and not distal hand movement per se. We can also partially compare our results to a sensing technique that did focus on distal hand movement: in [18] a finger-worn accelerometer was used to compare sensed finger angular velocity to a gold standard of a video-based tracking system. An average error of 11% was observed for unimpaired participants, but this value is difficult to directly compare because it is not based on a count of individual movements as in the present study.

### 4.3. Validating HAND Counts by Testing the Threshold Hypothesis of Hand Use after Stroke

The Threshold Hypothesis is an important idea in stroke rehabilitation research because it potentially provides a tangible rehabilitation goal: the patient must achieve a threshold level of hand function, and then they will use their hemiparetic hand at home. This home usage appears to trigger a “virtuous cycle” in which the usage produces further improvements in hand movement capability ([28]). In contrast, people with hand function that falls below a threshold enter a “vicious cycle” in which they lose hand function due to long-term disuse.

The Threshold Hypothesis was originally generated based on data from self-reports on a five-point scale (the Motor Activity Log [29]) for which people judged how much they use their limb in daily life. Here, we tested this hypothesis with a quantitative measure of distal hand use, i.e., HAND counts measured at home. Consistent with the Threshold Hypothesis, most individuals with low BBT scores generated HAND counts consistent with the measured rate of “false positives” due to environmental magnetic fields (i.e., ~200 counts/hour). The exceptions were two young, college students who showed somewhat higher HAND counts even with low BBT scores. One possibility is that these participants had increased daily transit and movement between classrooms due to attending college, which may have increased the rate of false positives caused by environmental magnetic fields. Another possibility is that they found more situations/needs to use their hand because of their college-related occupations. Determining the effect of age, commuter habits, and occupation on HAND counts is an important direction for future research.

Consistent with the Threshold Hypothesis, there was a steady increase in hand use intensity as participants’ BBT score increased beyond about 30. A BBT score of 30 is about half the normal score. In Schweighofer’s original work identifying the Threshold Hypothesis, subjects reported home arm activity scores on the Motor Activity Log between 2 (“Rarely—Sometimes used the weaker arm but did the activity most of the time with the stronger arm”) and 3 (“Half pre-stroke—Used the weaker arm about half as much as before the stroke”) when their functional score on the 5-point Functional Ability Scale was in the range 2.5 to 3.2. That is, a 50% functional score predicted the start of the use of the hand at home. The present study confirms this rule of thumb with a different functional scale and a sensor-based measurement of hand use.

This result should be compared to a recent result by Franck et al. [30] that acquired bilateral wrist accelerometry readings in 76 subacute stroke patients at home. They measured hours of active use of the impaired arm relative to the hours of active use of the non-dominant arms of unimpaired persons and plotted it against the UEFM, an impairment-based rather than functionally oriented scale. Active use of the impaired arm began to rise at an UEFM score between 50 and 60 (out of a total possible of 66), although the “threshold” was less distinct than in the present study. An UEFM score between 50 and 60 corresponds well to a BBT score of around 30–40 [25]. Thus, again, the rule of thumb for the Threshold Hypothesis appears to be that half-normal functional capability triggers real-world use.

### 4.4. Limitations and Future Work

Although the Manumeter increases the portability and social acceptability of a hand tracking device compared to current alternatives such as goniometers and cameras, it still requires users to wear two devices—the watch and the ring. This may be cumbersome for more cognitively impaired participants and future work on further reducing the required hardware is warranted.

The HAND algorithm had some crosstalk when arm-only exercises and the walking test were performed. More advanced data processing techniques could potentially be applied to further reduce these erroneous counts. For example, accelerometry data could be incorporated to support distinguishing hand movements from walking and arm movements. Slow hand movements were another source of inaccuracy. We do not know how often people perform slow, small hand movements at home, but more impaired persons would be expected to exhibit slower, smaller hand movements. Thus, it seems likely that the HAND algorithm undercounts movements for those with lower motor capability and further investigation is needed to understand this error, and to quantify the effects of functional and impairment measures to the accuracy of the algorithm.

Another potential source of inaccuracy results from interference with electromagnetic fields generated from small electronics and home appliances (for example, a laptop or cellphone). This problem is somewhat mitigated by the fact that these devices would need to move relative to the Manumeter for miscounts to happen. This movement is most likely to happen when the Manumeter (i.e., the participant’s hand) is moving relative to the object when true positives are more likely.

Using a stronger magnetic ring could help mitigate both inaccuracy issues described above by increasing the signal-to-noise ratio. Another way to improve the accuracy of the system is to use the already included accelerometer data in combination with magnetometry, because even distal hand and finger movements can cause peaks of acceleration in a wrist-worn device. Future experiments should also collect Manumeter data in the wild while also obtaining a ground truth (e.g., using a head-mounted camera [16]) to further optimize the parameters of the algorithm and validate it.

Another limitation of this study regards the robotic simulator used to validate the HAND algorithm, which only emulates MCP joint movements focused on a single degree for finger flexion and extension. Although it was not formally tested, it is likely that the HAND algorithm will not accurately recognize isolated abduction/adduction movements of the fingers alone (e.g., spreading the fingers apart). However, during daily activities, it would be expected that these movements would typically not happen in isolation, but in combination with the finger flexion/extension movements that are detected with the HAND algorithm. Nevertheless, further validation with human hands of different sizes, moving at a variety of controlled speeds will be included in future work.

### 4.5. Potential for Practical Application in Stroke Rehabilitation

We designed the HAND algorithm to be able to provide real-time feedback on hand activity to stroke survivors, to promote increased use of the hand, which has been postulated to be therapeutic through a virtuous cycle, as described above. We envision a practical scenario of application in which the user dons the Manumeter each morning and then tries to achieve a target number of HAND counts by the end of the day, much as the user of a pedometer typically tries to achieve 10,000 steps (or another goal) each day. In this scenario, the Manumeter will provide real-time feedback of HAND counts through a built-in display. We envision using the display to implement simple “games on the wrist” to motivate bursts of hand movement during the day. Indeed, we recently completed a randomized controlled trial of the effect of such HAND count feedback on hand use and recovery, and are in the process of analyzing those results.

## Figures and Tables

**Table 1:** Stroke survivors’ demographics for the HAND algorithm (± for standard deviation).

	Group 1 (*n* = 9)	Group 2 (*n* = 20)
Age	68 ± 9	57 ± 15
Gender (Male [M]/Female [F])	6 M/3 F	16 M/4 F
Time since stroke (months)	30 ± 23	40 ± 33
Side of hemiparesis (Right [R]/Left [L])	4 R/5 L	12 R/10 L
Type of stroke (Ischemic [I]/Hemorrhagic [H])	6 I/3 H	12 I/10 H
Box and Blocks Test (# blocks/60 s)	27 ± 20	21 ± 18
Fugl-Meyer Upper Extremity Score (out of 66)	43 ± 15	40 ± 13

## Data Availability

The data presented in this study are available on request from the corresponding author. The data are not publicly available due to privacy.
